# Chemical Constituents of Three *Allium* Species from Romania

**DOI:** 10.3390/molecules18010114

**Published:** 2012-12-21

**Authors:** Laurian Vlase, Marcel Parvu, Elena Alina Parvu, Anca Toiu

**Affiliations:** 1 Department of Pharmaceutical Technology and Biopharmaceutics, Faculty of Pharmacy, “Iuliu Hatieganu” University of Medicine and Pharmacy, 12 Ion Creanga Street, Cluj-Napoca 400010, Romania; Email: laurian.vlase@yahoo.com; 2 Department of Biology, Faculty of Biology and Geology, “Babes-Bolyai” University, 42 Republicii Street, Cluj-Napoca 400015, Romania; 3 Department of Pathophysiology, Faculty of Medicine, “Iuliu Hatieganu” University of Medicine and Pharmacy, 3 Victor Babes Street, Cluj-Napoca 400012, Romania; Email: parvualinaelena@yahoo.com; 4 Department of Pharmacognosy, Faculty of Pharmacy, “Iuliu Hatieganu” University of Medicine and Pharmacy, 12 Ion Creanga Street, 400010 Cluj-Napoca, Romania; Email: ancamaria_toiu@yahoo.com

**Keywords:** *Allium* species, alliin, allicin, polyphenolic compounds, phytosterols

## Abstract

The aim of this work was to study the chemical composition of *Allium obliquum* L., *A. senescens* L. subsp. *montanum* (Fries) Holub, and *A. schoenoprasum* L*.* subsp. *schoenoprasum*. Sulphur-containing compounds analysis was performed by an LC-MS method, the identification and quantification of polyphenolic compounds through a HPLC-UV-MS method, and the presence of five sterols was simultaneously assessed by HPLC-MS-MS. Alliin was identified only in *A. obliquum* and *A. senescens* subsp. *montanum* extracts, whilst allicin was present in all extracts, with higher amounts in *A. schoenoprasum* and *A. obliquum*. The pattern of phenol carboxylic acids shows the presence of *p*-coumaric and ferulic acids in all species. Isoquercitrin was identified in *A. obliquum* and *A. schoenoprasum*, and rutin in *A.* *senescens* subsp. *montanum* and *A. schoenoprasum*. Luteolin and apigenin were identified only in *A. obliquum*. All three species contain glycosides of kaempferol and quercetol. β-Sitosterol and campesterol were identified in all species. The results obtained showed significant differences in the composition of the three *Allium* species.

## 1. Introduction

Many epidemiological studies have suggested that certain natural foods could prevent the development of different diseases. Garlic and onion have a variety of pharmacological effects, including chemopreventive activity and tumor cell growth inhibition [1,2,3]. Previous studies showed that many *Allium* plants other than *A. sativum* and *A. cepa* are of great importance due to their uses as flavoring agents, antioxidants, fragrance and therapeutics [1]. The antioxidant activity of *Allium* species is due to a variety of sulphur-containing compounds and their precursors, but it is also related to other bioactive compounds such as polyphenols, dietary fiber and microelements [2].

Allicin is a flavour component of garlic (*Allium sativum*) which is formed due to the hydrolysis of alliin when the garlic tissue is damaged. To evaluate the quality of garlic and garlic products, it is important to consider all the precursors and the biological active substances present [3,4,5]. A decomposition product of allicin has hypocholesterolaemic activity [5]. Ajoene (a secondary degradation product of alliin) inhibits platelet aggregation by altering the platelet membrane via an interaction with sulphydryl groups [5]. Antimicrobial activity is well documented for garlic, and antifungal activity is more effective than nystatin (allicin is thought to be the main active component by inhibition of lipid synthesis). *In vitro* antiviral activity was attributed to allicin and its derivatives, and alliin has antihepatotoxic activity *in vitro* and *in vivo* [4,5].

Polyphenols are bioactive substances widely distributed in natural products. They have been reported to have multiple biological properties, such as antioxidant, antimutagenic, antibacterial, antiviral and anti-inflammatory activities [6,7]. Medicinal plants rich in polyphenols can retard the oxidative degradation of lipids and improve the quality and nutritional value of food [8].

The sterols occur in a large segment of plant species; both yellow and green vegetables contain appreciable quantities [9]. Phytosterols have demonstrated the capability to block the uptake of cholesterol (to which they are structurally related) and also facilitate its excretion from the body [9].

The most common phytosterols in natural products are β-sitosterol, stigmasterol, and campesterol. Sterols can reduce the atherosclerotic risk and offer protection against cardiovascular diseases [10]. They decrease the risks of breast, prostate and colon cancer [11,12]. Furthermore, phytosterols have anti-inflammatory and immunomodulatory properties [13]. All phytosterols in the human body come exclusively from the diet, as they cannot be synthesized by humans. More than 95% of total phytosterol dietary intake is represented by β-sitosterol, stigmasterol and campesterol [10].

The *Allium* L. *genus* includes more than 400 species that are widespread around the World. The antibacterial and antifungal properties of *Allium* sp. were demonstrated for *A. sativum*, *A. porrum* [14], *A. cepa* [15], *A. ascalonicum* [16], *A. fistulosum* [17], *A. minutiflorum* [18], *A. neapolitanum* [19], *A. obliquum* [4], *A*. *senescens* ssp. *montanum* [20], and *A. ursinum* [21]. The ethnobotanical data from Romania mention 32 wild and cultivated species of *Allium* L. [22]. *A. schoenoprasum* L*.* (chive) is a herbaceous perennial plant grown for its leaves which are used for both culinary and medicinal purposes. Chives have a beneficial effect on the circulatory system by lowering the blood pressure, and they have antimicrobial activity, especially antifungal, and antioxidant properties. The pharmacological effects are due to diallyl sulfides (diallyl monosulfide, diallyl disulfide, diallyl trisulfide, diallyl tetrasulfide), flavonoids, vitamin C, and carotenoids [3,23]. *A. obliquum* L. is an edible plant and a very rare perennial which is found in Romania in a single location, on limestone rocks in Turda Georges. As a wild species it can also be found in Central Asia and Siberia. *A. senescens* L. subsp. *montanum* (Fries) Holub is a wild species that occurs in the mountain areas of Central and Submediterranean Europe [22]. In traditional medicine, *A*. *senescens* ssp. *montanum* is used for its hypocholesterolemic, and digestive and circulatory system tonic effects [24].

To increase our understanding of the pharmacological and nutraceutical activities of *Allium* species, further comprehensive study of its nutrients, especially alliin, allicin, polyphenolic compounds and phytosterols, is essential. We employed a rapid, highly accurate and sensitive HPLC method assisted by MS detection for the simultanous determination of polyphenols in plants [25,26,27], and a newly developed LC-CIS-MS/MS method for the quantitative analysis of allicin and its precursor, alliin, in natural products [28].

The most frequent method used to determine sterols in plants is GC coupled with different detectors; there are several publications on HPLC-MS identification of phytosterols in vegetable oils and less in plant extracts [29,30,31,32]. Although numerous studies have been carried out for qualitative and quantitative determination of sterols in natural products, rather limited investigations have been conducted on phytosterols from some *Allium* species [33,34,35].

This is the first report of a simple, accurate and rapid HPLC-MS-MS method for identification and quantification of sterols from three species of *Allium*. The method is based on a previous published method [32], with some modification: the change of chromatographic column and mobile phase. Because the chemical composition of *Allium* species from Romania has been insufficiently studied, the aim of this work was to bring new data on sulphur-containing compounds, polyphenols and sterols on three *Allium* species: *A. obliquum* L., *A. senescens* L. subsp. *montanum* (Fries) Holub, and *A. schoenoprasum* L*.* subsp. *schoenoprasum*.

## 2. Results and Discussion

### 2.1. The Analysis of Polyphenols

**Figure 1:**
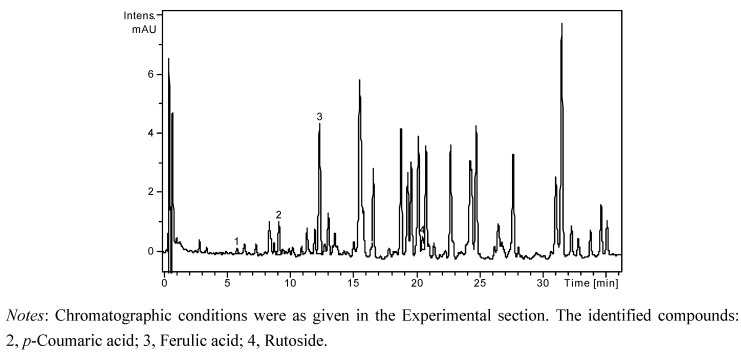
HPLC chromatogram of a non-hydrolysed sample of *A. senescens* subsp. *montanum* (A2 N).

**Figure 2:**
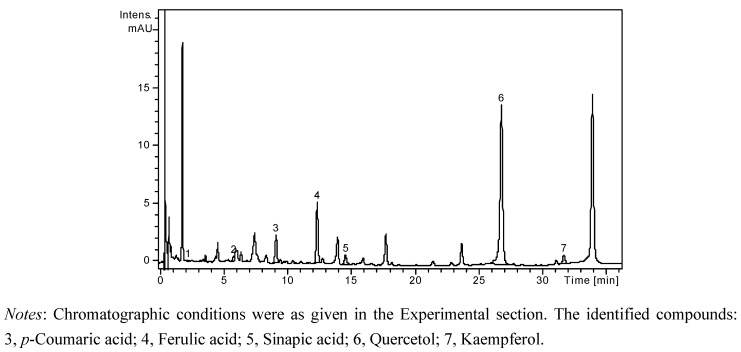
HPLC chromatogram of a hydrolysed sample of *A. senescens* subsp. *montanum* (A2 H).

**Figure 3:**
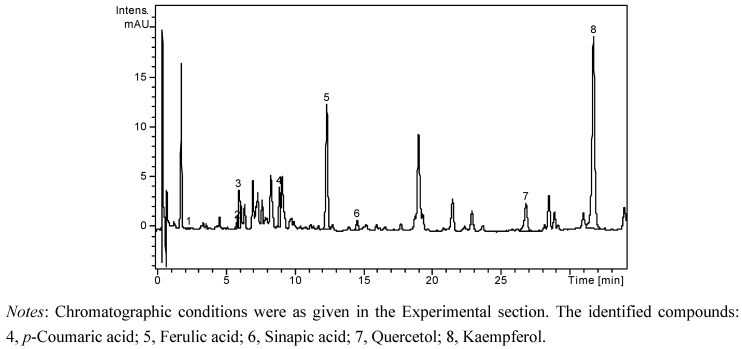
HPLC chromatogram of hydrolysed sample of *A. schoenoprasum* L*.* subsp. *schoenoprasum* (A3 H).

A high performance liquid chromatographic (HPLC) method has been developed for the determination of nineteen phenolic compounds (eight phenolic acids, four quercetin glycosides, and seven flavonol and flavone aglycones) from natural products. The simultaneous analysis of different classes of polyphenols was performed by a single column pass, and the separation of all examined compounds was carried out in 35 min. In order to obtain more accurate data on flavonoid glycosides and aglycones concentration, and to estimate the nature of hydrolysed compounds, each sample was analyzed before and after acid hydrolysis. The concentrations of identified polyphenolic compounds in all samples before and after acid hydrolysis are presented in Table 1. The HPLC chromatogram of a non-hydrolysed sample of *A. senescens* subsp. *montanum* (A2 N) is presented in Figure 1, the HPLC chromatogram of a hydrolysed sample of *A. senescens* subsp. *montanum* (A2 H) is presented in Figure 2, and the HPLC chromatogram of a hydrolysed sample of *A. schoenoprasum* L*.* subsp. *schoenoprasum* (A3 H) is presented in Figure 3.

**Table 1:** The polyphenolic compounds content of *Allium* species (μg/100 g vegetal product).

Sample	A1 ^N^	A1 ^H^	A2 ^N^	A2 ^H^	A3 ^N^	A3 ^H^
*p*-Coumaric acid	29.90 ± 0.21	85.16 ± 0.76	50.88 ± 0.75	125.99 ± 1.47	149.59 ± 1.05	163.71 ± 1.35
Ferulic acid	54.38 ± 0.54	343.54 ± 1.83	205.15 ± 1.69	226.96 ± 1.93	188.06 ± 1.51	542.33 ± 1.93
Sinapic acid	-	98.22 ± 0.88	-	48.99 ± 0.71	88.87 ± 0.67	44.91 ± 0.39
Isoquercitrin	123.38 ± 1.72	-	-	-	363.78 ± 1.89	-
Rutoside	-	-	51.60 ± 0.85	-	128.95 ± 1.55	-
Quercitrin	-	-	-	-	-	-
Quercetol	-	39.67 ± 0.27	-	993.90 ± 2.63	58.38 ± 0.62	200.48 ± 1.74
Luteolin	172.35 ± 1.56	280.74 ± 1.67	-	-	-	-
Kaempferol	-	35.80 ± 0.24	-	62.37 ± 0.74	129.83 ± 1.03	1563.46 ± 2.96
Apigenin	149.03 ± 1.03	277.06 ± 1.81	-	-	-	-

^N^ non-hydrolysed sample; ^H^ hydrolysed sample. Values are the mean ± SD (n = 3).

*p*-Coumaric acid and ferulic acid were identified in all ethanolic extracts. *A. senescens* was the richest species in ferulic acid (205.15 μg/100 g), and *A. obliquum* contains higher quantities of *p*-coumaric acid, both before and after hydroysis (149.59 μg/100 g, and 163.71 μg/100 g, respectively). Sinapic acid was present only in *A. schoenoprasum* before hydrolysys, and in all species after hydrolysis.

The pattern of flavonoids indicates large differences between the three *Allium* species, they can be used as potential taxonomic markers in order to distinguish the plants: isoquercitrin was identified in *A. obliquum* (123.38 μg/100 g), and *A. schoenoprasum* (363.78 μg/100 g), whereas rutin in *A. senescens* subsp. *montanum* (51.60 μg/100 g) and *A. schoenoprasum* (128.95 μg/100 g).

Luteolin and apigenin were determined only in *A. obliquum*, before (172.35 μg/100 g, and 149.03 μg/100 g, respectively), and after acid hydrolysis (280.74 μg/100 g, and 277.06 μg/100 g, respectively). Kaempferol and quercetol were present in both non-hydrolysed and hydrolysed sample of *A. schoenoprasum*, and only in hydrolysed extracts of *A. obliquum* and *A. senescens* subsp. *montanum*. The richest species in quercetol derivatives was *A. senescens* (993.90 μg/100 g), and in kaempferol derivatives was *A. schoenoprasum* (1563.46 μg/100 g).

We analyzed the polyphenols from three *Allium* species: *A. obliquum*, *A. senescens* subsp. *montanum*, and *A. schoenoprasum* subsp. *schoenoprasum*. The simultaneous determination of wide range of polyphenolic compounds was performed using a rapid, highly accurate and sensitive HPLC method assisted by mass spectrometry detection, and the comparative study showed large differences between the three *Allium* species. Considering the broad-spectrum therapeutic potential of polyphenols [36,37], further studies are needed to improve medicinal uses of *Allium* species from Romania.

### 2.2. The Analysis of Sulphur-Containing Compounds

In the literature there are analytical methods reporting detection of allicin by UV at 220 nm [38,39], but frequent interferences may appear at this wavelength because of the lack of selectivity, which can lead to measurement errors.

**Figure 4:**
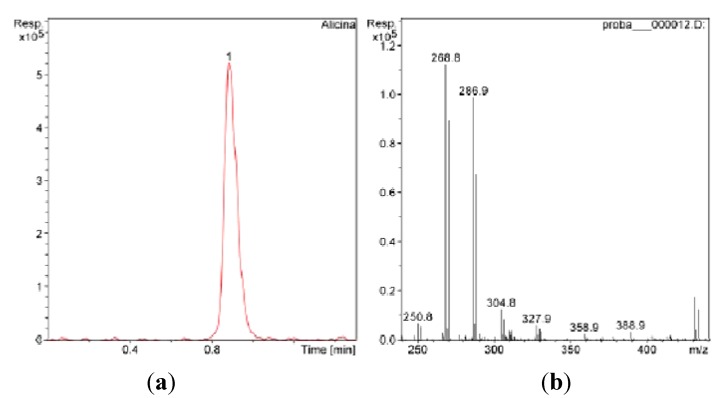
(**a**) The LC-MS chromatogram of allicin from *A. schoenoprasum* extract; (**b**) MS-MS spectra of allicin.

The sulphur-containing compounds have the ability to form adduct complexes with some transitional metals. The complex has an electric charge and it can be analyzed by mass spectrometry with electrospray ionization. In order to obtain selectivity in quantitative determination of allicin by LC-MS, we used the adduct complex formed by allicin and the silver ion for quantification [28]. The peak of allicin was observed at R_T_ = 0.9 min (Figure 4).

**Figure 5:**
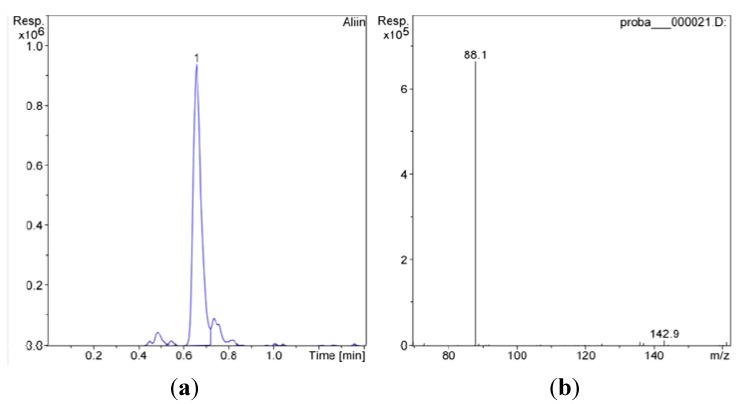
(**a**) The chromatogram of *A*. *obliquum* extract; (**b**) MS-MS spectra of alliin.

The chromatogram of *Allium obliquum* (A1) extract and MS-MS spectra of alliin are presented in Figure 5.

The results obtained for quantification of allicin and alliin in the *Allium* species extracts are presented in Table 2. The extracts prepared at room temperature (R extracts) have a lower allicin content than the extracts prepared by heating at 60 °C (C extracts), because higher temperature extraction transforms alliin into allicin.

**Table 2:** The content in alliin (mg/100 g vegetal product) and allicin (mg/100 g vegetal product) of *Allium* species extracts.

Species	Alliin	Allicin (R extracts)	Allicin (C extracts)
*A. obliquum* (A1)	13.65 ± 0.23	272.80 ± 1.62	426.62 ± 1.88
*A. senescens* (A2)	5.23 ± 0.19	82.47 ± 0.55	435.93 ± 1.64
*A. schoenoprasum* (A3)	N.D.	320.0 ± 1.71	947.22 ± 2.01

*Notes*: R, prepared at room temperature; C, prepared by heating. Values are the mean ± SD (n = 3). N.D., not determined.

Alliin was identified only in *A. senescens* subsp. *montanum* (A2) and *A. obliquum* (A1) extracts obtained by the repercolation method at room temperature, and the highest quantity was found in the former. Allicin was determined in all extracts obtained at room temperature, with the higher amounts in *A. schoenoprasum* and *A. obliquum*.

The allicin contents were higher in extracts obtained by heating than in those prepared at room temperature, because alliin and its derivatives were transformed into allicin under the working conditions (947.22 mg/100 g in *A. schoenoprasum*, 435.93 mg/100 g in *A. senescens*, and 426.62 mg/100 g in *A. obliquum*). The richest species in alliin was *A. obliquum* and the allicin content was higher in *A. schoenoprasum*.

### 2.3. The Analysis of Phytosterols

Under the proposed chromatographic conditions, retention times of the five analysed sterols were: 3.2 min for ergosterol, 3.9 min for brassicasterol, 4.9 min for stigmasterol and campesterol (co-elution) and 5.7 min for β-sitosterol. The ions monitorized in the MS method are presented in Table 3. Because in the ionization conditions all sterols have lost a water molecule, the ions detected by the spectrophotometer are always in the form [M−H_2_O+H]^+^.

**Table 3:** Characteristic ions of standard sterols in full scan and specific ions used in quantification.

Compound	R_T_ (min)	M	M−H_2_O+H^+^	Specific ions for identification Ion [M-H_2_O+H^+^] > Ions from spectrum
Ergosterol	3.2	396	379	379 > 158.9; 184.9; 199; 213; 225; 239; 253; 295; 309; 323
Brassicasterol	3.9	398	381	381 > 201.3; 203.3; 215.2; 217.3; 241.2; 255.3; 257.4; 271.1; 297.3; 299.3
Stigmasterol	4.9	412	395	395 > 255; 297; 283; 311; 241; 201
Campesterol	4.9	400	383	383 > 147; 149; 161; 175; 189; 203; 215; 229; 243; 257
β-Sitosterol	5.7	414	397	397 > 160.9; 174.9; 188.9; 202.9; 214.9; 243; 257; 287.1; 315.2

In the process of mass-spectrometry analysis, the psedudo-mollecular ions of sterols (379 for ergosterol, 381 for brassicasterol, 395 for stigmasterol, 383 for campesterol and 397 for β-sitosterol) have been fragmented, and based on their daughter ions from the MS spectrum the extracted chromatograms of each compound were constructed. The method can also be applied for quantitative determination because the intensity of ions in the mass spectrum is proportional to the concentration of the substance in the sample.

In order to quantify the five sterols from Allium species extracts, we have constructed the extracted chromatograms for each compound, taking into account the intensity of major ions in the mass spectrum (Table 3).

Calibration curves were obtain from standard solutions at different concentration levels, selected as representative of the range of concentration in the sample. Regression analysis of various concentrations of standard solutions (0.08–8 µg/mL) gave good correlation coefficients for the calibration curves of sterols.

β-Sitosterol and campesterol were quantified in all species: *A. obliquum* (211.30 mg/100 g vegetal product, and respectively 12.90 mg/100 g vegetal product), *A. senescens* (72.40 mg/100 g vegetal product, and respectively 25.5 mg/100 g vegetal product), and *A. schoenoprasum* (25.09 mg/100 g vegetal product, and respectively 7.21 mg/100 g vegetal product). The richest species in β-sitosterol was *A. obliquum*, and campesterol was found in higher concentration in *A. senescens*. This is the first report for determination of phytosterols content in *A. obliquum* L., *A. senescens* L. subsp. *montanum* (Fries) Holub, and *A. schoenoprasum* L*.* subsp. *schoenoprasum*.

## 3. Experimental

### 3.1. General

General Apparatus and Chromatographic Conditions: an Agilent 1100 HPLC Series system (Agilent, Santa Clara, CA, USA) was used, coupled with an Agilent Ion Trap SL mass spectrometer equipped with an electrospray or APCI ion source.

### 3.2. Chromatographic Conditions for the Analysis of Polyphenolic Compounds

The experiment was carried out using an Agilent 1100 HPLC Series system equipped with degasser, binary gradient pump, column thermostat, autosampler and UV detector. The HPLC system was coupled with an Agilent 1100 mass spectrometer (LC/MSD Ion Trap VL). For the separation, a reverse-phase analytical column was employed (Zorbax SB-C18 100 × 3.0 mm i.d., 3.5 μm particle); the work temperature was 48 °C. The detection of the compounds was performed on both UV and MS mode. The UV detector was set at 330 nm until 17.5 min, then at 370 nm. The MS system operated using an electrospray ion source in negative mode. The chromatographic data were processed using ChemStation and DataAnalysis software from Agilent.

The mobile phase was a binary gradient prepared from methanol and solution of 0.1% acetic acid (v/v). The elution started with a linear gradient, beginning with 5% methanol and ending at 42% methanol, for 35 min; isocratic elution followed for the next 3 min with 42% methanol. The flow rate was 1 mL/min and the injection volume was 5 μL.

The MS signal was used only for qualitative analysis based on specific mass spectra of each polyphenol. The MS spectra obtained from a standard solution of polyphenols were integrated in a mass spectra library. Later, the MS traces/spectra of the analysed samples were compared to spectra from library, which allows positive identification of compounds, based on spectral mach. The UV trace was used for quantification of identified compounds from MS detection. Using the chromatographic conditions described above, the polyphenols eluted in less than 40 min (Table 4). Four polyphenols cannot be quantified in current chromatographic conditions due overlapping (caftaric acid with gentisic acid and caffeic acid with chlorogenic acid). However, all four compounds can be selectively identified in MS detection (qualitative analysis) based on differences between their molecular mass and MS spectra. For all compounds, the limit of quantification was 0.5 µg/mL, and the limit of detection was 0.1 µg/mL. The detection limits were calculated as minimal concentration producing a reproductive peak with a signal-to-noise ratio greater than three. Quantitative determinations were performed using an external standard method. Calibration curves in the 0.5–50 μg/mL range with good linearity (R2 > 0.999) for a five point plot were used to determine the concentration of polyphenols in plant samples [25,26].

**Table 4:** Retention times (R_T_) of polyphenolic compounds (min).

Peak no.	Phenolic compound	*m/z*	R_T_ ± SD	Peak no.	Phenolic compound	*m/z*	R_T_ ± SD
1.	Caftaric acid	311	3.54 ± 0.05	11.	Rutoside	609	20.76 ± 0.15
2.	Gentisic acid	153	3.69 ± 0.03	12.	Myricetin	317	21.13 ± 0.12
3.	Caffeic acid	179	6.52 ± 0.04	13.	Fisetin	285	22.91 ± 0.15
4.	Chlorogenic acid	353	6.43 ± 0.05	14.	Quercitrin	447	23.64 ± 0.13
5.	*p*-Coumaric acid	163	9.48 ± 0.08	15.	Quercetol	301	27.55 ± 0.15
6.	Ferulic acid	193	12.8 ± 0.10	16.	Patuletin	331	29.41 ± 0.12
7.	Sinapic acid	223	15.00 ± 0.10	17.	Luteolin	285	29.64 ± 0.19
8.	Cichoric acid	473	15.96 ± 0.13	18.	Kaempferol	285	32.48 ± 0.17
9.	Hyperoside	463	19.32 ± 0.12	19.	Apigenin	279	39.45 ± 0.15
10.	Isoquercitrin	463	20.29 ± 0.10				

*Note*: SD, standard deviation.

### 3.3. Chromatographic Conditions for the Analysis of Alliin

The separation of alliin was made using a Zorbax SB-C18 100 mm × 3.0 mm i.d., 3.5 μm column (Agilent Technologies, Darmstadt, Germany). The mobile phase consisted in 100% ammonium acetate 1mM in water, isocratic elution, flow 1 mL/min. The mass spectrometer operated in positive multiple reaction monitoring mode (MRM), using and electrospray ion source and nitrogen as nebulising and dry gas. The nebuliser was set at 70 psi, the dry gas flow was 12 L/min at 350 °C. The apparatus was set to record the transition *m/z* 178 > *m/z* 88, which is specific to alliin. The retention time of alliin in above described conditions was 0.64 min.

### 3.4. Chromatographic Conditions for the Analysis of Allicin

The separation of allicin was made using a Synergi Polar 100 mm × 2.0 mm i.d., 4 µm column (Phenomenex, Torrance, CA, USA). The mobile phase consisted in 100% ammonium acetate, 1 mM in water, isocratic elution, flow 0.6 mL/min. A silver nitrate solution 1 mM in water was added post column, with a flow of 10 µL/min. The mass spectrometer operated in positive MRM mode, using an electrospray ion source and nitrogen as nebulising and dry gas. The nebuliser was set at 60 psi, the dry gas flow was 12 L/min at 350 °C. The apparatus was set to record the transition *m/z* (449+451) > *m/z* (269; 271; 287; 289), specific to allicin-silver complex. The retention time of allicin in the above described conditions was 0.9 min.

### 3.5. Chromatographic Conditions for the Analysis of Phytosterols

Compounds were separated using a Zorbax SB-C18 reversed-phase analytical column (100 × 3.0 mm i.d., 5 μm particle) fitted with a guard column Zorbax SB-C18, both operated at 40 °C. Sterols were separated under isocratic conditions using a mobile phase consisting of 10:90 (v/v) methanol and acetonitrile. The flow rate was 1 mL/min and the injection volume was 5 μL. Mass spectrometry analysis was performed on an Agilent Ion Trap 1100 VL mass spectrometer with atmospheric pressure chemical ionization (APCI) interface. The instrument was operated in positive ion mode. Operating conditions were optimized in order to achieve maximum sensitivity values: gas temperature (nitrogen) 325 °C at a flow rate of 7 L/min, nebulizer pressure 60 psi and capillary voltage −4,000 V.

The full identification of compounds was performed by comparing the retention times and mass spectra with those of standards in the same chromatographic conditions. To avoid or limit the interference from background, the multiple reactions monitoring analysis mode was used instead of single ion monitoring (e.g., MS/MS instead of MS).The Agilent ChemStation (vA09.03) and DataAnalysis (v5.3) software were used for the acquisition and analysis of chromatographic data.

### 3.6. Chemicals

Standards: chlorogenic acid, *p*-coumaric acid, caffeic acid, rutin, apigenin, quercetin, isoquercitrin, hyperoside, kaempferol, quercetol, myricetol, fisetin, alliin, β-sitosterol, brassicasterol, stigmasterol, campesterol and ergosterol from Sigma (St. Louis, MO, USA), ferulic acid, sinapic acid, gentisic acid, patuletin, luteolin from Roth (Karlsruhe, Germany), cichoric acid, caftaric acid from Dalton (Toronto, ON, Canada), allicin from Allicin International (East Sussex, UK), with a purity ≥98.0%. Methanol of HPLC analytical-grade, acetonitrile of HPLC analytical-grade, ammonium acetate of HPLC analytical-grade, silver nitrate of HPLC analytical-grade, chloroform, *n*-hexane, potassium hydroxide of analytical-grade and hydrochloric acid of analytical-grade were purchased from Merck (Darmstadt, Germany). Methanolic stock solutions (100 mg/mL) of the flavonoid standards were prepared and stored at 4 °C, protected from daylight. They were appropriately diluted with double distilled water before being used as working solutions. Methanolic stock solutions (4 mg/mL) of alliin and allicin were prepared and stored at 4 °C, protected from daylight. They were appropriately diluted with double distilled water before being used as working solutions. Chloroformic stock solutions (1 mg/mL) of the phytosterol standards were prepared and stored at 4 °C, protected from daylight. Before being used as working solutions, they were appropriately diluted with acetonitrile. Distilled, deionised water was produced by a Direct Q-5 Millipore (Millipore SA, Molsheim, France) water system.

### 3.7. Identification and Quantitative Determinations

The detection and quantification of polyphenols was made in UV assisted by mass spectrometry detection. Due peak overlapping, four polyphenol-carboxylic acids (caftaric, gentisic, caffeic, chlorogenic) were determined only based on MS spectra, whereas for the rest of compounds the linearity of calibration curves was very good (R^2^ > 0.998), with detection limits in the range of 18 to 92 ng/mL. The detection limits were calculated as minimal concentration producing a reproductive peak with a signal-to-noise ratio greater than three. Quantitative determinations were performed using an external standard method; retention times were determined with a standard deviation ranging from 0.04 to 0.19 min (Table 4). For all compounds, the accuracy was between 94.1.3% and 105.3%. Accuracy was checked by spiking samples with a solution containing each polyphenol in a 10 μg/mL concentration. In all analyzed samples the compounds were identified by comparison of their retention times and recorded electrospray mass spectra with those of standards in the same chromatographic conditions.

The identification of sterols was performed by comparing the retention times and mass spectra with those of standards in the same chromatographic conditions. To avoid or limit the interference from background, the multiple reactions monitoring analysis mode was used instead of single ion monitoring (e.g., MS/MS instead of MS). The Agilent ChemStation (vA09.03) and DataAnalysis (v5.3) software were used for the acquisition and analysis of chromatographic data. Linearity of calibration curves was very good (R^2^ > 0.998), with detection limits in the range of 69 to 3,312 ng/mL for ergosterol, 62 to 2,952 ng/mL for brassicasterol, 59 to 2,808 ng/mL for campesterol, 136 to 6,528 ng/mL for stigmasterol, and 132 to 6,336 ng/mL for β-sitosterol. The calibration curve of alliin standard was linear between 149.6–7,480.0 ng/mL and the calibration curve of allicin standard was linear between 18–864 µg/mL.

### 3.8. Plant Material and Preparation of Extracts

Fresh *Allium obliquum* L.(A1), *A. senescens* L. subsp. *montanum* (Fries) Holub (A2), and *A. schoenoprasum* L*.* subsp. *schoenoprasum* (A3) herba (leaves, stems and flowers fragments of 0.5 to 1 cm) was used for extraction with 70% ethanol (Merck, Bucuresti, Romania) in the Mycology Laboratory of Babes-Bolyai University, Cluj-Napoca, Romania, by a modified Squibb repercolation method [40]. Briefly, three successive applications of the same solvent were repercolated to the plant material. In each percolator, plant material (150 g in the first, 90 g in the second, 60 g in the third percolator) was moistened with the solvent, macerated for two days and then percolated at a rate of about 4 to 6 drops per min for each 100 g of raw material. The first percolated fractions from each percolator were saved and the next fractions were poured in the next percolator. Then, saved fractions (60 mL from the first one, 90 mL from the second one and 150 mL from the third one) were mixed and the resulting extract was 1:1 (w:v) [4,17].

All plants were identified and voucher specimens (CL 659564-A1, CL 659563-A2, CL 659561-A3) were deposited at the Herbarium of “A. Borza” Botanical Garden, “Babes-Bolyai” University of Cluj-Napoca, Romania.

In order to obtain more accurate data on flavonoid glycosides and aglycones concentration, each sample was analyzed before and after acid hydrolysis. Extractive solution (2 mL) was treated with 2M hydrochloric acid (2 mL) and ascorbic acid solution (0.2 mL, 100 mg/mL), and the mixtures were heated at 80 °C on a water bath for 30 min, ultrasonicated for 15 min, and heated for another 30 min at 80 °C. During the heating, methanol (1 mL) was added to the extraction mixture every 10 min, in order to ensure the permanent presence of methanol. The mixtures were centrifuged at 4,000 rpm and the solutions were diluted with distilled water in a 10 mL volumetric flask and filtered through a 0.45 µm filter before injection.

## 4. Conclusions

We analyzed the polyphenols from three *Allium* species: *A. obliquum*, *A. senescens* subsp. *montanum*, and *A. schoenoprasum* subsp. *schoenoprasum*, and we completed the literature data with new information concerning the polyphenolic substances from *Allium* species*.* The simultaneous determination of a wide range of polyphenolic compounds was performed using a rapid, highly accurate and sensitive HPLC method assisted by mass spectrometry detection. The contents of alliin and allicin were also determined, showing the transformation of alliin and its derivatives into allicin by heating. The analysis of phytosterols from the three *Allium* species was performed for the first time, and we quantified β-sitosterol and campesterol in ethanolic extracts. The comparative study showed large differences, both qualitative and quantitative, between the three *Allium* species.
